# Caffeine intake enhances bowel recovery after colorectal surgery: a meta-analysis of randomized and non-randomized studies

**DOI:** 10.1007/s13304-024-01847-x

**Published:** 2024-05-03

**Authors:** Sascha Vaghiri, Dimitrios Prassas, Stephan Oliver David, Wolfram Trudo Knoefel, Andreas Krieg

**Affiliations:** 1 Department of Surgery (A), Heinrich-Heine-University and University Hospital Duesseldorf, Duesseldorf, Germany; 2 Department of Surgery, Katholisches Klinikum Essen, Philippusstift, Teaching Hospital of Duisburg-Essen University, Huelsmannstrasse 17, 45355 Essen, Germany; 3 Department of General and Visceral Surgery, Thoracic Surgery and Proctology, University Hospital Herford, Medical Campus OWL, Ruhr University Bochum, Schwarzenmoorstr. 70, 32049 Herford, Germany

**Keywords:** Caffeine intake, Coffee intake, Colorectal surgery, Postoperative ileus, Postoperative complications

## Abstract

Postoperative ileus (POI) after colorectal surgery is a major problem that affects both patient recovery and hospital costs highlighting the importance of preventive strategies. Therefore, we aimed to perform a systematic analysis of the effects of postoperative caffeine consumption on bowel recovery and surgical morbidity after colorectal surgery. A comprehensive literature search was conducted through September 2023 for randomized and non-randomized trials comparing the effect of caffeinated versus non-caffeinated drinks on POI by evaluating bowel movement resumption, time to first flatus and solid food intake, and length of hospital stay (LOS). Secondary outcome analysis included postoperative morbidity in both groups. After data extraction and inclusion in a meta-analysis, odds ratios (ORs) for dichotomous variables and standardized mean differences (SMDs) for continuous outcomes with 95% confidence intervals (CIs) were calculated. Subgroup analyses were performed in cases of substantial heterogeneity. Six randomized and two non-randomized trials with a total of 610 patients were included in the meta-analysis. Caffeine intake significantly reduced time to first bowel movement [SMD −0.39, (95% CI −0.66 to −0.12), *p* = 0.005] and time to first solid food intake [SMD −0.41, (95% CI −0.79 to −0.04), *p* = 0.03] in elective laparoscopic colorectal surgery, while time to first flatus, LOS, and the secondary outcomes did not differ significantly. Postoperative caffeine consumption may be a reasonable strategy to prevent POI after elective colorectal surgery. However, larger randomized controlled trials (RCTs) with homogeneous study protocols, especially regarding the dosage form of caffeine and coffee, are needed.

**Supplementary Information:**

The online version contains supplementary material available at 10.1007/s13304-024-01847-x.

## Introduction

Postoperative ileus (POI), defined as a temporary disruption of intestinal motility is a common and concerning phenomenon especially following colorectal surgery with documented POI rates ranging from 10.2% to 19% [1, 2]. POI is not only associated with patient discomfort and increased susceptibility to ileus-related complications but may also be a reason of delayed hospital discharge, resulting in additional economic burden for healthcare providers [3]. Many factors have been reported in the literature to be associated with prolonged cessation of bowel activity after colorectal surgery including smoking history, open approach, preoperative albumin levels, fluid management, and intra-abdominal complications [4–7]. Implementation of fast-track protocols with concurrent attention to these adjustable perioperative variables have been successful strategies for overcoming POI [8]. However, the quest for cost-effective and sufficient preventive measures to further reduce surgical morbidity and costs continues. Coffee and caffeinated drinks are among the most popular drinks being consumed worldwide. Coffee consists of a complex mixture of more than 1000 physiological and bioactive compounds, with anti-oxidative, anti-inflammatory and anti-cancer effects [9, 10]. In addition to natural constituents, the complex biochemical process of roasting and coffee preparation, such as the Maillard reaction, can alter the final composition and the degree of physiological interaction [11, 12]. Nevertheless, coffee consumption is associated with many health benefits in cardiovascular, metabolic, and neurodegenerative diseases and reduces the risk of all-cause mortality [13–15]. Interestingly, the mechanism of action of coffee on the brain-gut axis with its propulsive effects is not fully understood [16]. While the beneficial effect of coffee consumption on postoperative bowel recovery after gynecologic surgery and cesarean section has been consistently demonstrated in randomized controlled trials (RCTs) [17–19], there are still conflicting results regarding coffee and caffeine intake in colorectal surgery, especially with regard to bowel motility [20–23]. Thus, the primary objective of this meta-analysis was to accurately evaluate the impact of caffeine and coffee consumption on postoperative outcomes after colorectal surgery, with a special focus on bowel recovery, as a potential cost-effective, easily accessible, and practical strategy for POI prevention.

## Methods

The meta-analysis was conducted according to the current Preferred Reporting Items for Systematic Reviews and Meta-Analyses (PRISMA) checklist [24] and the Cochrane Handbook for Systematic Reviews of Interventions [25].

### Search strategy

A systematic database search was conducted independently by two authors (S.V., and D.P.) in Pubmed (Medline), and the Cochrane Central trials register up to September 2023. There were no time or language restrictions. The following key search terms were used in combination with the Boolean operators AND or OR: “coffee”, “caffeine”, “drinks”, “postoperative ileus”, “colorectal surgery”, and “intestinal transit”. In addition, the reference list of the retrieved studies, systematic reviews or conference proceedings was screened to identify potentially relevant citations for the analysis. Each selected abstract and study was again independently assessed by two reviewers for eligibility and inclusion in the meta-analysis. Disagreements were resolved by discussion and consensus. If differences remained, a third author (S.O.D.) was consulted.

### Selection criteria

All original studies comparing postoperative outcomes of caffeinated drink consumption (e.g. coffee, juice; defined as the intervention group) versus decaffeinated coffee, water, or tea (control) in open or minimally-invasive colorectal surgery for benign and malignant diseases were considered eligible. To be included in the meta-analysis, studies had to report on at least one of the following outcomes: gastrointestinal (GI) motility parameters (time to first bowel movement, time to first flatus, time to first oral diet intake), use of adjunctive laxatives, and surgical morbidity. Studies without colorectal resection (e.g. only rectopexy or only small bowel surgery) were excluded. In the case of duplicate or overlapping articles published by the same institution and authors, the most recent study was selected for inclusion.

### Data extraction

All relevant data were entered independently by two authors (S.V., and D.P.) into an electronic data extraction sheet from articles meeting the inclusion criteria. Disagreements were discussed and resolved by consensus or reassessment by a third author (S.O.D.). The following data were extracted from each included study:

1. Study characteristics: first author, year and country of publication, study design and randomization, enrollment period, number of patients in each group [ITT (intention-to -treat)/PP (per-protocol)], type of surgical procedure, inclusion and exclusion criteria, fast-track compliance, study- protocols, intervention and comparator group definitions, and study endpoints.
2. Demographic and patient related information: Age, sex, BMI (body mass index), ASA (American Society of Anesthesiologists) classification, medical comorbidities, preoperative coffee consumption, smoking history, malignant or benign disease indicated for surgery.
3. Surgical data: access route (open, laparoscopic, robotic), duration of surgery, site/extent of surgical resection and type of bowel anastomosis.
4. GI motility and recovery outcomes, length of hospital stay (LOS), overall postoperative morbidity and major complications according to the Clavien-Dindo classification [26].

### Outcome measures

The primary outcomes of this study were POI-related variables including time to first documented bowel movement, time to first flatus, time to first solid diet intake and LOS. In addition, the use of laxatives, nasogastric tube re-insertion, overall and major morbidity, re-operation rate, anastomotic leak, and mortality were parameters of our secondary outcome analysis.

### Quality and certainty assessment

The risk of bias of the six included randomized trials was assessed using the RoB 2 criteria [27]. Briefly, this recommended tool categorizes randomized trials into low to high risk of bias based on signaling questions derived from five potential bias domains (randomization process, deviations from the intended intervention, missing outcome data, measurement of the outcome, and selection of the reported results). In parallel, the risk of bias of the two remaining non-randomized studies was evaluated using the ROBINS-I tool [28], which also classifies studies from low to critical risk of bias according to the assessment of seven different bias domains. The authors independently evaluated the risk of bias of each included study. Disagreements were discussed and resolved by consensus. The revised AMSTAR 2 instrument [29] was used to critically appraise this meta-analysis. The level of evidence for important primary and secondary outcomes was classified into four categories (high, moderate, low, and very low) according to GRADE (The Grading of Recommendations, Assessment, Development, and Evaluation) [30].

### Statistical analyses

Statistical analysis was performed using RevMan software (version 5.3. Copenhagen: The Nordic Cochrane Centre, The Cochrane Collaboration, 2014) according to the recommendations of the Cochrane Collaboration guidelines. Pairwise meta-analyses were performed. Summary treatment effect estimates with 95% confidence intervals (CIs) were calculated for each outcome of interest. Odds ratios (ORs) and the Mantel–Haenszel method were used for dichotomous outcomes. Standardized mean differences (SMDs) were calculated to analyze continuous outcomes. The methods by Luo et al. [31] and Wan et al. [32], or the Box-Cox (BC) method of McGrath et al. [33] were applied to estimate the sample mean and standard deviation from studies providing a summary set of median, quartile range, and sample size. In case of missing values, the study authors were contacted directly to provide the data if possible. Continuous values were expressed in hours (time to first bowel movement, time to first flatus and solid diet intake), and in days (length of hospital stay). The degree of heterogeneity among the included studies was interpreted as follows after applying the Cochrane Q test (chi-square test; Chi2) and measuring inconsistency (I^2^): 0–40% low heterogeneity and may not be important, 30%-60% moderate heterogeneity, 50–90% substantial heterogeneity, 75–100% high heterogeneity. Note that starting with moderate heterogeneity, the significance of the obtained I^2^ value is dependent on the size and direction of the effects and the power of evidence for heterogeneity (e.g., p-value of the Chi2 test or the I^2^ confidence interval) [25]. If heterogeneity was low or moderate (I^2^ < 50%), summary estimates were calculated using a fixed-effects method. Otherwise, if I^2^ > 50%, the random-effects model was used. In cases of substantial heterogeneity, the source of heterogeneity was further investigated using one-way sensitivity and subgroup analyses. Subgroup analyses were performed according to surgical approach (open versus minimally-invasive), site of resection (right versus left colectomy), and type of coffee administered (caffeinated versus decaffeinated coffee) to test the stability of the meta-analysis when appropriate. Publication bias tests and funnel plots were not performed due to the small number of studies included in the meta-analysis. A p-value of < 0.05 was considered significant.

## Results

### Study and patient characteristics

**Fig. 1:**
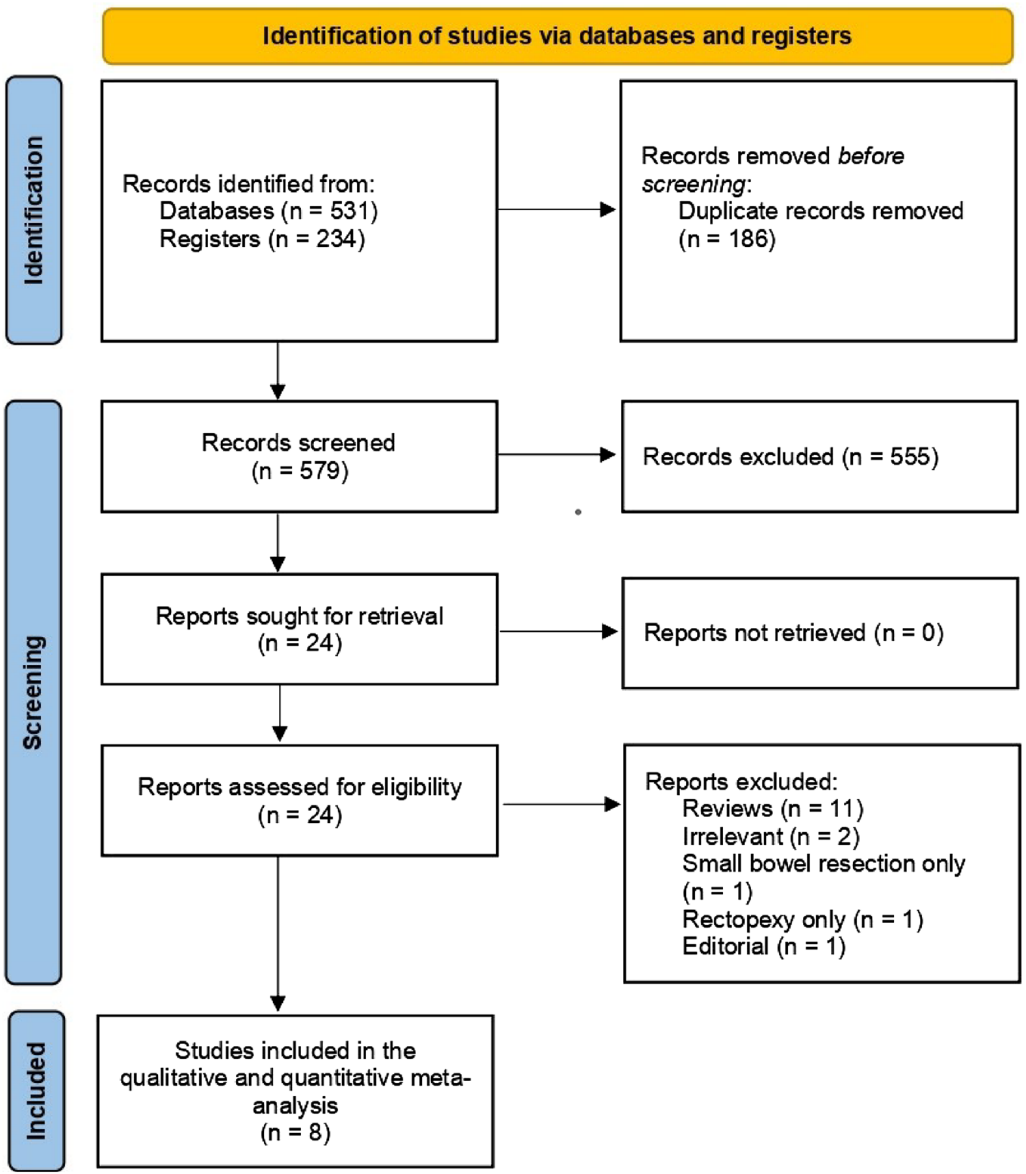
PRISMA diagram of study identification and selection for review analysis

Our initial systematic database search identified 765 records. After removing duplicates and irrelevant articles, 24 full-text articles were assessed for eligibility. Based on the predefined inclusion criteria, eight studies [20–23, 34–37] of elective colorectal surgery were eligible for our final meta-analysis (Fig. 1).

Six of the included studies were RCTs [20–23, 34, 36], while two studies did not use randomization [35, 37]. Five studies originated from Europe [20, 22, 34–36], two from the Middle East [21, 37], and one from North America [23]. In all but one study [34], patients were assigned on a single-center basis. A total of 610 patients were enrolled from March 2010 to June 2022 (caffeine: n = 286, control: n = 324). All studies used caffeinated drinks (mostly coffee) as the main intervention of interest. In two studies, caffeine was given in apple-flavored water and cornstarch drinks [21, 22]. In all studies, caffeinated drinks (single dose of 100–150 ml) were administered three times daily from the same day of surgery until the second postoperative day. Interestingly, two studies compared caffeinated coffee with decaffeinated coffee drink [20, 23]. In both study groups the majority of cases were performed minimally-invasively (caffeinated drinks: 81.47%, control: 82.41%). One study included only open surgery [35]. The site of resection included both right and left-sided colectomies in seven studies [21–23, 34–37]. Only left-sided resections were reported in the study by Dulskas et al. [20]. In addition, four patients underwent rectopexy procedure [23]. Adherence to fast-track protocols was mentioned in four studies [20, 23, 34, 36]. A thoracic epidural catheter was used for analgesia in two studies [22, 34]. A detailed summary of the study, patient, and surgical characteristics are presented in Tables 1, 2, and 3.

**Table 1:** Study characteristics and protocols

Author	Year	Origin	Study period	Study design	Sample size	Surgical procedure	Fast-track protocol	Epidural analgesia	Intervention	Comparator	Intervention start
Müller et al. [34]	2012	Germany	Mar 2010–Mar 2011	Multi-center, open label, RCT	79 (ITT)/71 (PP)	Elective open/lap. colon resection	Yes	Yes	Coffee (100 ml)/3x daily (coffee capsules)	Warm water (100 ml)	POD 1
Dulskas et al. [20]	2015	Lithuania	Jan 2013–Dec 2014	Single-center, prospective, RCT	96 (ITT)/90 (PP)	Elective left-sided colectomy	Yes	NS	caffeinated coffee (100 ml)/3× daily, (coffee capsules)	Decaffeinated coffee (100 ml)/3× daily, (capsules), water (100 ml)	POD 1
Piric et al. [35]	2015	Bosnia and Herzegovina	2013–2014	Single-center, retrospective	60 (ITT)/58 (PP)	Elective open colorectal resection	NS	NS	Coffee (100 ml)/3× daily, (instant coffee)	Tea (100 ml)	POD 2
Hasler-Gehrer et al. [36]	2019	Switzerland	Sep 2014–Dec 2016	Single-center, prospective, open-label, RCT	115 (ITT)/96 (PP)	Elective lap. colorectal resection	Yes	No	Coffee (150 ml)/3× daily, (coffee beans)	Tea (150 ml)	POD 1
Parnasa et al. [21]	2021	Israel	Nov 2017–Mar 2019	Single-center, prospective, double blinded RCT	63 (ITT)/58 (PP)	Elective lap. colorectal resection	NS	No	caffeine citrate (100 mg)/apple-flavored water (50 ml)/3× daily	Apple-flavored water (50 ml)	POD 1
Abbassi et al. [22]	2022	Switzerland	Oct 2015–Aug 2020	Single-center, placebo-controlled, double blinded, RCT	60 (ITT)/27 (PP)	Elective lap. colorectal resection	NS	Yes	Caffeine (100/200 mg)/3× daily, dispensed into capsules with corn starch	Corn starch capsules	Same day of surgery
Bildirici et al. [37]	2022	Turkey	Nov 2018–Jun 2019	Single-center, prospective, non-randomized	60 (ITT)/51 (PP)	Elective lap. colorectal surgery	NS	NS	Coffee (100 ml)/3× daily, (instant coffee)	Water (100 ml)	Same day of surgery
Nasseri et al. [23]	2023	USA	Dec 2016–Jun 2021	Single-center, prospective, RCT	102 (ITT)/99 (PP)	Elective minimally-invasive colorectal surgery	Yes	No	Caffeinated coffee (116 ml)/3× daily	Decaffeinated coffee 3x daily (116 ml), warm water (116 ml)	POD 1

*ITT* intention-to-treat, *NS* not stated, *POD* postoperative day, *PP* per-protocol, *RCT* randomized controlled trial

**Table 2:** Demographic data and characteristics of the included patients

Author	Groups	No. of patients	Age (years) mean/SD	Gender (M/F)	BMI (kg/m^2^) mean/SD	ASA score	Preoperative coffee consumption	Smoking history	Operative indication	
									Malignant	Benign
Müller et al. [34]	Coffee	40	62 ± 12	25/15	NS	ASA I–II 29 ASA III 11	35	5	23	17
	Control	39	59 ± 15	19/20	NS	ASA I–II 27 ASA III 12	34	9	22	17
Dulskas et al. [20]	Caffeinated coffee	30	67.3 ± 6.8*	16/14	NS	NS	NS	5	30	0
	Decaffeinated coffee	30	62.4 ± 10.8*	16/14	NS	NS	NS	3	30	0
	Control	30	66.3 ± 9.1*	16/14	NS	NS	NS	3	30	0
Piric et al. [35]	Coffee	28	63.57 ± 1.969	17/11	NS	ASA I–II 19 ASA III–IV 9	NS	3	25	3
	Control	30	62.67 ± 3.082	17/13	NS	ASA I–II 22 ASA III–IV 8	NS	1	28	2
Hasler-Gehrer et al. [36]	Coffee	56	64.06 ± 12.935	31/25	27.31 ± 4.869	ASA I–II 43 ASA III 9	NS	15	23	33
	Control	59	65.62 ± 11.94	28/31	26.75 ± 3.21	ASA I–II 45 ASA III 6	NS	11	29	30
Parnasa et al. [21]	Caffeine citrate	30	56.90 ± 12.77	15/15	27.07 ± 4.33	ASA I-II 28 ASA III 2	NS	7	NS	NS
	Control	28	55.36 ± 15.48	14/14	28.15 ± 5.63	ASA I-II 25 ASA III 3	NS	6	NS	NS
Abbassi et al. [22]	Caffeine 200 mg	20	61.2 ± 7.0	9/11	26.7 ± 5.3	ASA I–II 18 ASA III 2	17	1	NS	NS
	Caffeine 100 mg	20	63.7 ± 8.8	15/5	25.7 ± 2.7	ASA I–II 20 ASA III 0	18	5	NS	NS
	Control	20	64.1 ± 12.9	11/9	26.5 ± 4.6	ASA I–II 19 ASA III 1	19	3	NS	NS
Bildirici et al. [37]	Coffee	25	58.72 ± 13.25	13/12	26.57 ± 4.75	NS	24	5	25	0
	Control	26	61.44 ± 12.3	17/9	26.15 ± 4.38	NS	18	2	26	0
Nasseri et al. [23]	Caffeinated coffee	37	59.5 ± 15.0	22/15	25.7 ± 5.1	ASA I-II 23 ASA III 14	NS	9	17	20
	Decaffeinated coffee	31	63.7 ± 14.7	19/12	29.6 ± 7.5	ASA I-II 16 ASA III 15	NS	5	22	9
	Control	31	61.6 ± 15.5	20/11	26.9 ± 5.5	ASA I-II 20 ASA III 11	NS	9	15	16

*ASA score* American Society of Anesthesiologists, *BMI* body max index, *NS* not stated, *SD* standard deviation

*Median (range)

**Table 3:** Operative characteristics

Author	Groups	Type of procedure/resection								Type of access	Type of anastomosis	Duration surgery (min) mean/SD
		Ileocecal	Right colectomy	Left colectomy	Sigmoid/recto-sigmoid	(Low) Anterior resection	Rectopexy	Subtotal/total colectomy	Segmental colectomy	Open/MIS	Hand-sewn/stapled	
Müller et al. [34]	Coffee	4	11	4	21	0	0	0	0	24/16	24/16	173 ± 56
	Control	6	15	5	13	0	0	0	0	24/15	24/15	183 ± 57
Dulskas et al. [20]	Caffeinated coffee	0	0	7	12	11	0	0	0	0/30	0/30	102 ± 37.2*
	Decaffeinated coffee	0	0	5	13	12	0	0	0	0/30	0/30	103 ± 42.5*
	Control	0	0	5	16	9	0	0	0	0/30	0/30	98.0 ± 35.2*
Piric et al. [35]	Coffee	0	5	6	10	7	0	0	0	28/0	6/22	139.3 ± 6.764
	Control	0	15	9	2	4	0	0	0	30/0	19/11	130.8 ± 6.798
Hasler-Gehrer et al. [36]	Coffee	0	15	8	30	3	0	0	0	1/55	15/41	160.35 ± 37.284
	Control	1	21	6	30	1	0	0	0	3/56	22/37	153.53 ± 37.994
Parnasa et al. [21]	Caffeine citrate	0	17	0		8	0	5	0	0/30	NS	NS
	Control	0	7	0		18	0	3	0	0/28	NS	NS
Abbassi et al. [22]	Caffeine 200 mg	0	0	0	17	1	0	0	2	0/20	NS	161.9 ± 49.0
	Caffeine 100 mg	0	2	0	15	3	0	0	0	0/20	NS	172.1 ± 61.0
	Control	0	3	1	16	0	0	0	0	0/20	NS	162.3 ± 45.6
Bildirici et al. [37]	Coffee	0	4	3	0	18	0	0	0	0/25	NS	234.61 ± 64.8
	Control	0	6	3	0	17	0	0	0	0/26	NS	240.0 ± 76.8
Nasseri et al. [23]	Caffeinated coffee	0	12	11	0	5				0/37	NS	NS
	Decaffeinated coffee	0	13	13	0	3	4	7	2	0/31	NS	NS
	Control	0	7	20	0	2				0/31	NS	NS

*MIS* minimally-invasive surgery, *NS* not stated

*Median (range)

### Study quality and risk of bias

**Fig. 2:**
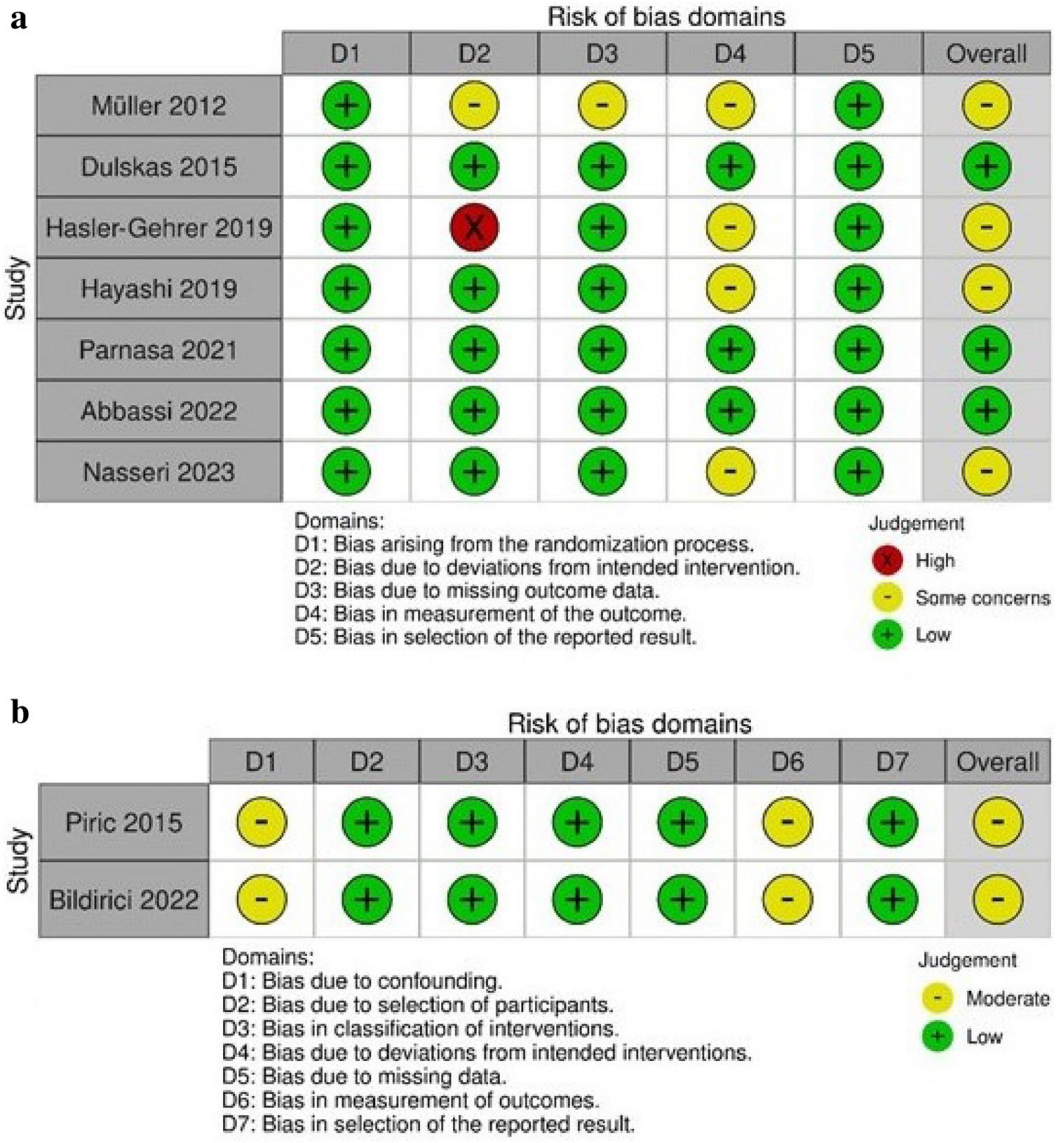
Risk of bias summary according to **a** RoB 2 **b** ROBINS-I

According to the RoB 2 criteria for randomized trials, the overall risk of bias was considered to be low in three RCTs, while some concerns were evident in the remaining three studies (Fig. 2a). The ROBINS-I tool assessment of the non-randomized studies showed an overall moderate risk of bias (Fig. 2b). The main limitations were that blinding of patients and outcome assessors was evident in only three studies [20–22]. In addition, the different proportions of right- and left-sided colectomies performed in the caffeine and control groups in four studies [21, 23, 35, 36] could lead to significant selection bias. The methodological quality of the present meta-analysis was determined as `high` using the AMSTAR 2 quality assessment tool.

### Primary outcome analysis

#### Time to first bowel movement

**Fig. 3:**
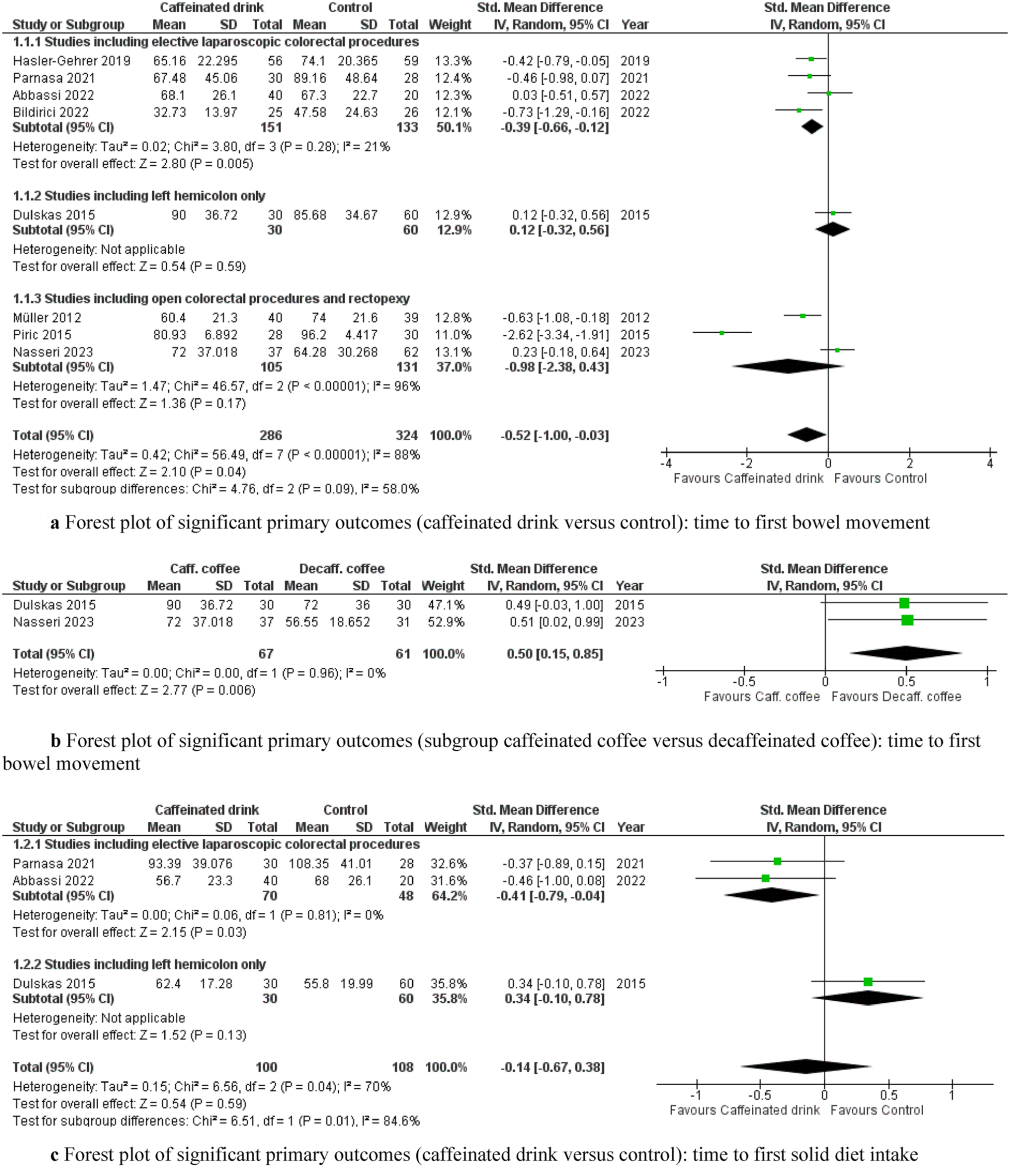

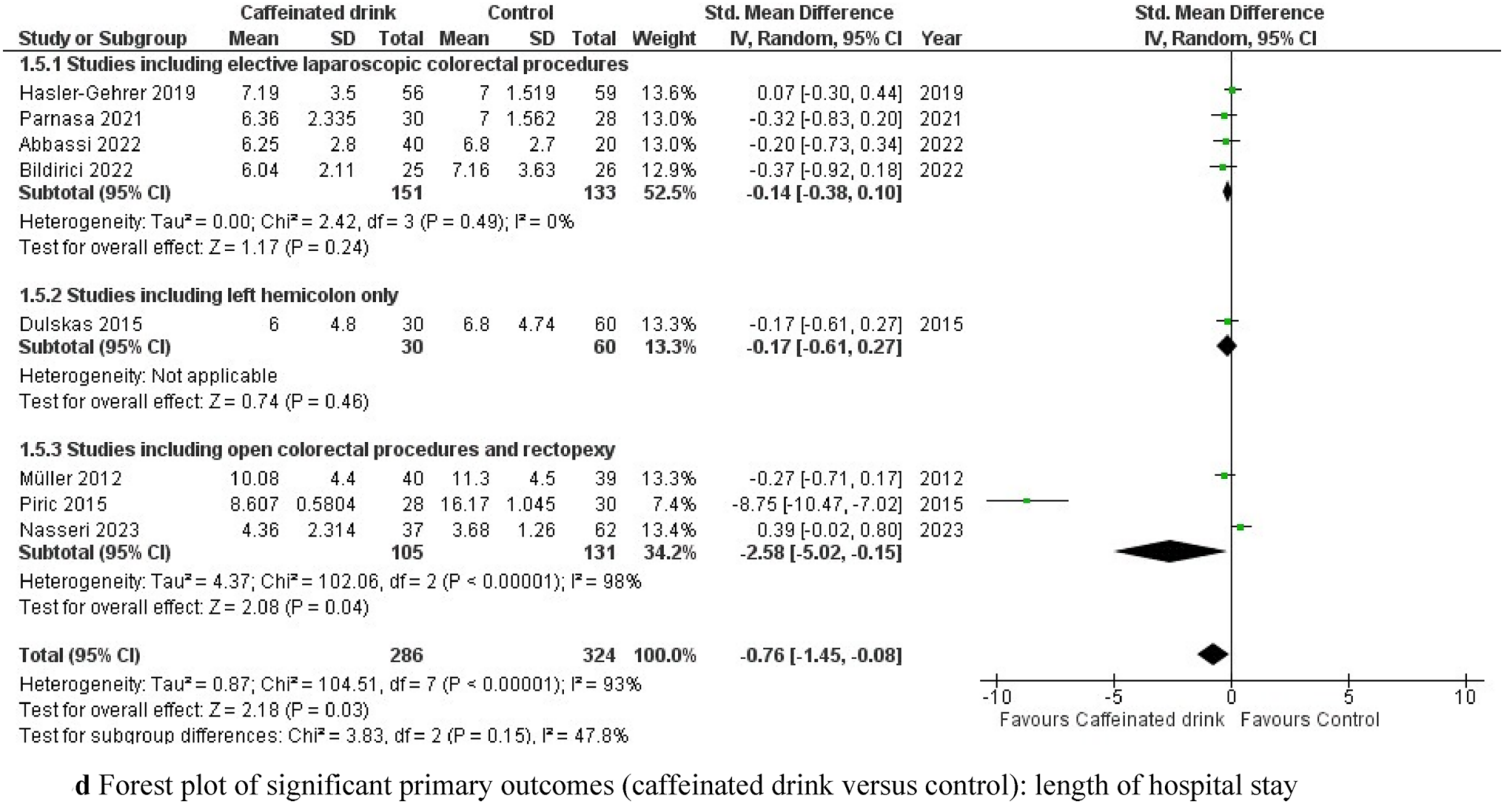
**a** Forest plot of significant primary outcomes (caffeinated drink versus control): time to first bowel movement. **b** Forest plot of significant primary outcomes (subgroup caffeinated coffee versus decaffeinated coffee): time to first bowel movement. **c** Forest plot of significant primary outcomes (caffeinated drink versus control): time to first solid diet intake. **d** Forest plot of significant primary outcomes (caffeinated drink versus control): length of hospital stay

Time to first recorded bowel movement was available in all included studies [20–23, 34–37] with a total of 610 patients. The consumption of caffeinated drinks resulted in a significant reduction in the time to first bowel movements compared to the control group [SMD −0.52, (95% CI −1.00 to −0.03), p = 0.04]. Of note, a significant level of heterogeneity was observed (I^2^ = 88%, Chi^2^ test: p < 0.00001). Importantly, subgroup analysis revealed that in studies including only elective laparoscopic colorectal procedures (both right- and left-sided) [21, 22, 36, 37], the results were reproducible [SMD −0.39, (95% CI −0.66 to −0.12), p = 0.005], but with a low level of heterogeneity (I^2^ = 21%, Chi^2^ test: p = 0.28). Thus, the source of heterogeneity was identified in the group of studies with open or non-colorectal resection procedures (I^2^ = 96%, Chi^2^ test: p < 0.00001) [23, 34, 35] (Fig. 3a). Interestingly, when comparing this outcome in the subgroup of caffeinated versus decaffeinated coffee [20, 23], restoration of first noticed bowel movement was significantly faster in patients receiving decaffeinated coffee [SMD 0.50, (95% CI 0.15–0.85), p = 0.006], (I^2^ = 0%, Chi^2^ test: p = 0.96) (Fig. 3b). The level of certainty of evidence based on the GRADE criteria was low (Table S1).

#### Time to first solid diet intake

Three studies [20–22] reported the time to first solid diet tolerance, including 208 patients, with no significant difference in the time to first food intake in both groups [SMD −0.14, (95% CI −0.67 to 0.38), p = 0.59]. The degree of heterogeneity was high (I^2^ = 70%, Chi2 test: p = 0.04). Of note, subgroup analysis demonstrated a faster resumption of solid food intake in studies reporting elective laparoscopic right- and left-sided colectomy [21, 22] [SMD −0.41, (95% CI −0.79 to −0.04), p = 0.03] with low heterogeneity (I^2^ = 0%, Chi^2^ test: p = 0.81) (Fig. 3c).

#### Time to first flatus

The time of first documented flatus was reported in six studies [20–23, 36, 37] with 473 patients. Our meta-analysis showed no statistically significant difference in first postoperative flatus between the caffeine and control groups [SMD −0.07, (95% CI −0.36 to 0.22), p = 0.64]. A moderate level of heterogeneity was observed (I^2^ = 58%, Chi^2^ test: p = 0.04) (Table 4).

**Table 4:** Primary and secondary non-significant outcomes

Outcomes	No. of included studies	No. of included patients		SMD/OR [95% CI]	P-value	Heterogeneity level	
		Caffeinated drinks	Control			I^2^ (%)	P-value
Primary							
Time to first flatus (hours)	6 [20–23, 36, 37]	218	255	−0.07 [−0.36–0.22]	0.64	58	0.04
Secondary							
Additive laxative use	3 [34–36]	124	128	0.64 [0.38–1.06]	0.08	26	0.26
NG-tube re-insertion	6 [21, 23, 34–37]	216	244	1.15 [0.61–2.19]	0.66	17	0.30
Re-operation	4 [21, 23, 34, 35]	135	159	0.42 [0.09–1.83]	0.25	0	0.69
Overall complications	6 [20, 22, 23, 34–36]	231	270	0.73 [0.47–1.13]	0.15	0	0.66
Severe complications (CD ≥ 3b)	4 [22, 23, 34, 36]	173	180	0.44 [0.17–1.10]	0.08	0	0.98
Anastomotic leak	3 [34–36]	124	128	0.42 [0.11–1.64]	0.21	30	0.23
Mortality	7 [20–23, 34–36]	261	297	0.14 [0.01–2.79]	0.20	NA	NA

*CD* Clavien-Dindo, *NG-tube* nasogastric tube, *NA* not applicable, *OR* odds ratio, *SMD* standardized mean difference

#### Length of hospital stay (LOS)

LOS was reported in all eight included studies [20–23, 34–37]. Postoperative caffeine consumption resulted in a significantly shorter hospital stay than in the control group [SMD −0.76, (95% CI −1.45 to −0.08), p = 0.03]. Notably, there was a substantial degree of heterogeneity between studies (I^2^ = 93%, Chi^2^ test: p < 0.00001). After subgroup analysis, this difference became non-significant [SMD −0.14, (95% CI −0.38 to 0.10), p = 0.24] in the subgroup of elective laparoscopic right- and left-sided colorectal studies [21, 22, 36, 37] with low heterogeneity (I^2^ = 0%, Chi^2^ test: p = 0.49). The source of heterogeneity was identified in the open and non-colorectal resection cohort [23, 34, 35] (I^2^ = 98%, Chi^2^ test: p < 0.00001) despite significant benefits of caffeine intake in this subgroup [SMD −2.58, (95% CI −5.02 to −0.15), p = 0.04] (Fig. 3d). According to GRADE, the level of evidence for this outcome was very low (Table S1).

### Secondary outcome analysis

The results of the secondary outcome meta-analyses indicated no statistically significant differences between the caffeine and control groups in terms of laxative use, nasogatric tube re-insertion, need of re-operation, overall complications, major complications (CD ≥ 3b), anastomotic leak, and mortality rates with a low level of heterogeneity (I^2^ between 0% and 30%) (Table 4).

## Discussion

The results of the current meta-analysis with eight included studies revealed, in contrast to the previously published literature [38], that postoperative caffeine intake accelerates bowel recovery after colorectal surgery, especially in the subgroup of patients undergoing elective minimally-invasive colorectal surgery with a low degree of heterogeneity. While the time to first bowel activity was significantly shorter in the caffeine group, there was no difference in the time to first solid diet tolerance in either the caffeine or control groups, although the subgroup analysis of elective minimally-invasive procedures suggested a significant benefit of postoperative caffeine intake in terms of oral diet resumption. As a result, the length of hospital stay was significantly shorter in the caffeine group. However, this benefit appeared to be relevant only in the cohort of open and non-colorectal procedures. To ensure homogenous groups, we distinguished between caffeinated drinks including coffee and drinks without caffeine such as decaffeinated coffee. Of note, in two of the included studies caffeine was dispensed in other drinks than coffee [21, 22], and two studies used decaffeinated coffee as control [20, 23]. Therefore, in our opinion, the arbitrary inclusion of caffeine and coffee in one group could introduce a risk of bias.

The development of postoperative ileus (POI), although to some extent considered a transient physiological response [39], is triggered by a complex neuro-immuno-inflammatory interaction [40, 41]. Preventive strategies are becoming increasingly important to avoid operation-related morbidities associated with postoperative ileus, thereby reducing hospital stay and healthcare costs [42]. Coffee consists of hundreds of bioactive compounds that undergo multiple modifications during the preparation process from the native bean to the final product, explaining the complexity of its action. Several components including caffeine, CGA (chlorogenic acid), melanoidins, and diterpenes, are associated with mucous secretion and gastrointestinal motor function [16]. Coffee consumption has been described to stimulate intestinal motility in healthy individuals [43] and after colorectal surgery [44] and small bowel resection [45]. The physiological effect of caffeine on intestinal activity is based on several mechanisms, including calcium-mediated vasodilation [46], vagus nerve stimulation [47], and gastrin release [48]. At the same time, the anti-inflammatory effect of chlorogenic acid by inhibiting tumor necrosis factor-α and interleukin-6 production results in less edema formation and pain relief [49, 50]. In fact, Piric et al. [35] were able to demonstrate significantly lower postoperative CRP (C-reactive protein) levels in the coffee group compared to the control group.

Interestingly, our subgroup analysis showed that decaffeinated coffee had a stronger effect on bowel movements than caffeinated coffee, as the resumption of the first documented bowel movement was earlier in patients who consumed decaffeinated coffee, suggesting that components other than caffeine may play a critical role in GI-tract motility [51]. Furthermore, it is hypothesized that the decaffeination process itself may result in the formation of more bioactive products [52].

Several limitations must be considered when interpreting the results; the included studies served a variety of coffee products (e.g. instant coffee, coffee/caffeine capsules) with different volumes ranging from 100–150 ml. This could not only lead to significant heterogeneity between studies, but also complicate the investigation of a dose–response relationship. Studies using tea as a control [35, 36] neglect the potential prokinetic effect of tea and its compounds on gastrointestinal motility [53, 54]. Remarkably, in all study protocols, the first coffee or caffeine administration was started in the postoperative period (the same day after surgery until second day). Based on pharmacokinetic principles, caffeine achieves its full effect at least 23 h after initiation [55], thus mitigating the potential impact on intestinal motility in the setting of postoperative ileus and recovery [56]. Another important methodological weakness was the lack of blinding of investigators and patients, as only three trials masked the investigators [20–22]. Blinding of the participating patients in a coffee or caffeine study is difficult due to the nature of the protocol. However, in two studies the taste of caffeine was neutralized by dispensing [21, 22]. The type of approach and the extent or side of resection may also significantly influence outcomes. It has been shown that patients undergoing open surgery and right-sided colectomy have a higher incidence of postoperative ileus [57, 58]. This is consistent with our observation showing a GI motility benefit of caffeine in the subset of studies using minimally-invasive approaches [21, 22, 36, 37]. In our meta-analysis two studies included open resections [34, 35], while in four studies the proportion of right- and left sided colectomy was not evenly distributed [21, 23, 35, 36]. Other important concerns include the relatively small and heterogeneous sample size (median 65.5 patients) with varying characteristics, lack of information on fast- track protocols [21, 22, 35, 37], and the use of epidural analgesia as an important preventive POI factor [59] mentioned in only two studies [22, 34]. Finally, in all studies investigating postoperative GI motility after abdominal surgery, there is a variable definition of ileus, which may limit the results presented. None of the studies included in our analysis used the recommended and evidence-based composite outcome measure GI-2 (time to tolerance of oral diet and passage of stool) [60].

## Conclusions

Postoperative caffeine consumption significantly reduces POI after colorectal surgery, especially when minimally-invasive approaches are used. Therefore, this simple, safe, and easily implemented measure could be incorporated into enhanced recovery programs. However, the limited level of evidence due to various bias concerns must be rigorously addressed by larger studies with uniform protocols to provide generalizable recommendations. Thus, additional high-quality prospective RCTs are needed to make a definitive statement.

### Supplementary Information

Below is the link to the electronic supplementary material.

Supplementary file1 (DOCX 27 KB)

## Data Availability

Not applicable.
