# Supplementary material for: Caffeine intake enhances bowel recovery after colorectal surgery: a meta-analysis of randomized and non-randomized studies
Source: Updates Surg. 2024 May 3;76(3):769–82. doi: 10.1007/s13304-024-01847-x (PMC11129976; doi:10.1007/s13304-024-01847-x)
Supplement: Supplementary file 1 — Supplementary file1 (DOCX 27 KB) [file 13304_2024_1847_MOESM1_ESM.docx]

Table S1 The GRADE Certainty assessment for the significant outcomes

| Outcomes | No. of studies | **No. of included patients** | | SMD [95 % CI] | **Quality assessment** | | | | | Quality |
| --- | --- | --- | --- | --- | --- | --- | --- | --- | --- | --- |
|  |  | Caffeinated drink | Control |  | Risk of bias^a^ | Inconsistency | Indirectness | Imprecision | Publication bias |  |
| Time to first bowel movement | 8 [20–23, 34–37] | 286 | 324 | -0.52 [-1.00 to -0.03] | Not serious | Serious | No indirectness | No imprecision | NA | Low |
| Length of hospital stay (LOS) | 8 [20–23, 34–37] | 286 | 324 | -0.76 [–1.45 to -0.08] | Not serious | Very serious | No indirectness | No imprecision | NA | Very low |

NA: not applicable, SMD: standardized mean difference ^a^ Risk of bias assessed using the ROBINS-I and RoB 2 tools.
